# A Semi-Automatic Dispenser for Solid and Liquid Food in Aquatic Facilities

**DOI:** 10.1089/zeb.2019.1733

**Published:** 2019-08-01

**Authors:** Raphaël Candelier, Alex Bois, Stéphane Tronche, Jéremy Mahieu, Abdelkrim Mannioui

**Affiliations:** ^1^ Laboratoire Jean Perrin, LJP, Centre National de la Recherche Scientifique, Sorbonne Université, Paris, France.; ^2^ Institut de Biologie Paris-Seine (IBPS), Aquatic Facility, Sorbonne Université, Paris, France.

**Keywords:** zebrafish, dispenser, automation, feeding

## Abstract

We present a novel, low-footprint and low-cost semi-automatic system for delivering solid and liquid food to zebrafish, and more generally to aquatic animals raised in racks of tanks. It is composed of a portable main module equipped with a contactless reader that adjusts the quantity to deliver for each tank, and either a solid food module or a liquid food module. Solid food comprises virtually any kind of dry powder or grains below 2 mm in diameter, and, for liquid-mediated food, brine shrimps (*Artemia salina*) and rotifers (*Rotifera*) have been successfully tested. Real-world testing, feedback, and validation have been performed in a zebrafish facility for several months. In comparison with manual feeding this system mitigates the appearance of musculoskeletal disorders among regularly-feeding staff, and let operators observe the animals' behavior instead of being focused on quantities to deliver. We also tested the accuracy of both humans and our dispenser and found that the semi-automatic system is much more reliable, with respectively 7-fold and 84-fold drops in standard deviation for solid and liquid food.

## Introduction

Since the pioneering work of Streisinger *et al.* published in 1981 on cloning homozygous diploid zebrafish,^1^ *Danio rerio* has rapidly grown as a model system in many different fields including embryo development, tissue regeneration, and neuroscience. In 2017, it has been estimated that more than 5 million zebrafish were used in more than 3.250 institutes spread across 100 countries.^2,3^ It is more common in laboratories than other well-established aquatic vertebrates like xenopus^4^ and medaka^5^ and, for instance, it is the second most common animal species used for research in Great Britain.^6^ As research on zebrafish has gained an impressive momentum in such a short time lapse, a large number of dedicated fish rooms have appeared. Other species like *Danionella translucida* (now amenable to brain-wide functional imaging in adults with cellular resolution^7^) and killifish (whose short lifespan, fecundity, and diapause of dried eggs make an ideal model for biogerontology^8^) also have a high potential for a rapid spread among research institutes in the future. Altogether, there has been and there will be a growing need for improving husbandry procedures in aquatic facilities, with very different scales ranging from a few hundred to hundreds of thousands animals.

Feeding is a fundamental task in any fish room, and an active area of research focuses on improving the nutritional quality of the food.^9,10^ Live food is generally preferable to purely artificial diets,^11,12^ as live feeds possess balanced nutritional profiles,^9^ are visually and chemically attractive to fish, and are highly digestible.^13^ It also contributes to the animal's welfare in captivity with the ability to actively hunt and express natural feeding behaviors.^14^ It is also mandatory according to the European regulation. However, only a few technical advances have been proposed on how food is actually delivered. It is traditionally performed manually with wash bottles for live food in liquid medium (e.g., *Artemia* nauplii, rotifers) and with various systems ranging from spoon-like tools to seed sowers for powders and granulates. Manual feeding raises serious issues though, with a clear lack of control over the delivered quantities and the appearance of musculoskeletal disorders among technicians. More specifically, wrist, elbow and shoulder tendinopathy are common among fish room staff, mainly because of the repetitive application of pressure on wash bottles. It may cause recurrent work stoppages, and in the most severe cases require steroid injections and surgery.

The only serious alternative to manual feeding is a fully-automated commercial solution, but it is extremely expensive, has a large footprint (which makes it difficult or impossible to install in small spaces, stand-alone racks or some fish rooms located in buildings that were not initially built for this purpose), processes very slowly (thus does not guarantee that microorganisms are still alive when delivered to the animals), delivers discrete quantities of food (which has limited accuracy), prevents access to the tanks during operation, and still requires food-filling and regular maintenance. In addition, breakdown—which is an inherent risk to every automated system—can have catastrophic consequences for both animals and the research associated.

Here we present an intermediate solution between manual and fully-automated systems, keeping the assets of both approaches while eliminating most of their drawbacks. Our semi-automatic food dispenser (SeAFooD) is battery-powered and portable with a low footprint, delivers dry solid or liquid-mediated food in a modular manner, displaces all the weight of liquid in a self-supporting reservoir on caster wheels, requires no specific gesture for triggering and remains under the operation of a human agent at all times. We quantified that microorganisms have a high or perfect survival rate while going through the dispenser and that survivors are as motile as control. The dispenser can deliver either fixed quantities, operator-controlled quantities or obtain information on the number of individuals in each tank *via* near-field communication (NFC) and automatically deliver the exact amount of food. The latter mode (1) has an accuracy down to the single-animal scale or below, (2) diminishes waste and improves water quality, (3) allows for custom diets, and (4) let the operator focus on animal behavior during the feeding process. The whole system is low-cost and has been built with standard tools anyone can find in a FabLab (e.g., 3D printer, laser-cutter, soldering iron). Finally, it has been tested in a medium-scale zebrafish platform for several months and had a very positive impact on the staff health since all staff members observed a decrease in forearm pains during this period.

## Materials and Methods

### General description

**FIG. 1.**
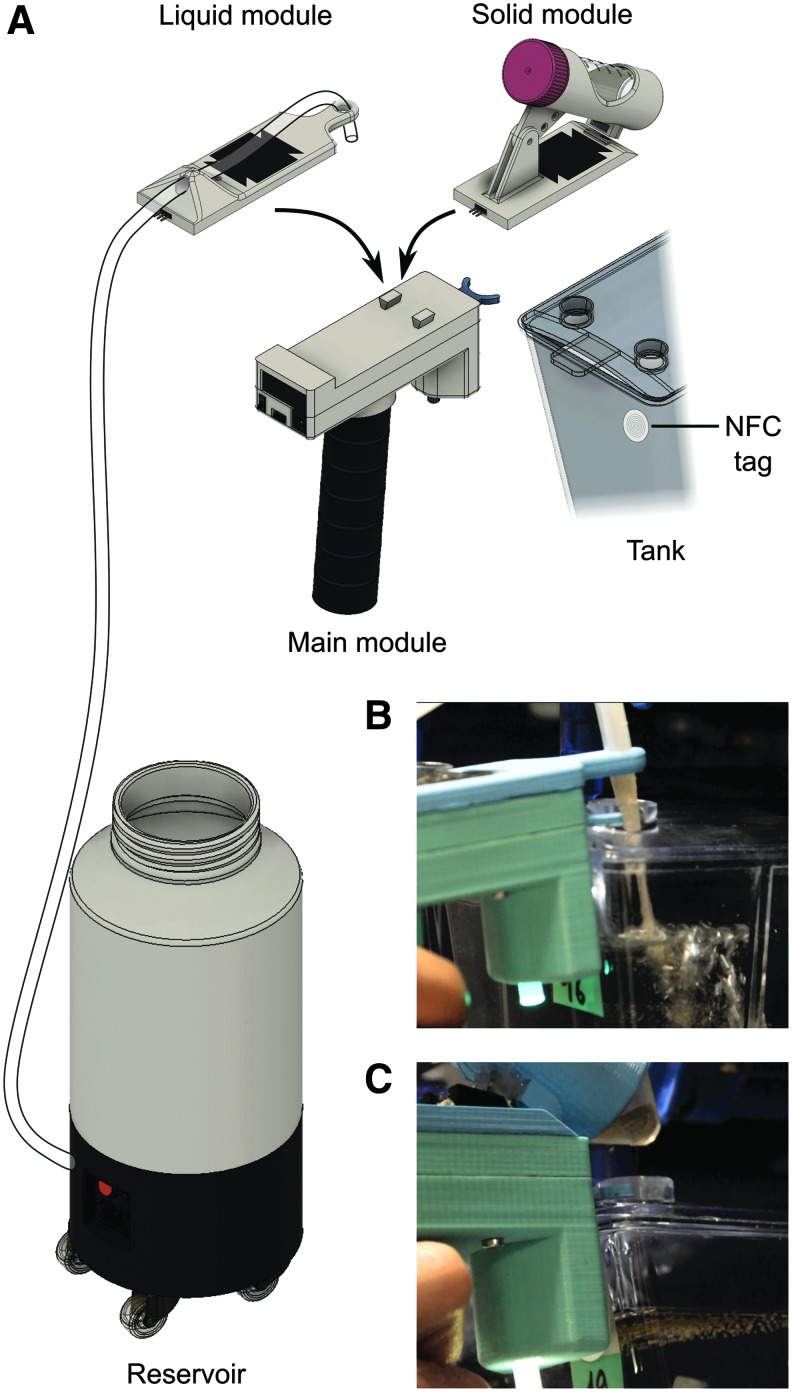
The semi-automatic food dispenser. **(A)** Scheme of the system. The main module can host either the liquid food or solid food module to form a functional assembly. The solid food module directly hosts 50 mL tubes of powder while the liquid food module has an external 8 L reservoir mounted on caster wheels. **(B)** Picture of the liquid food assembly during delivering. **(C)** Picture of the solid food assembly during delivering.

**FIG. 2.**
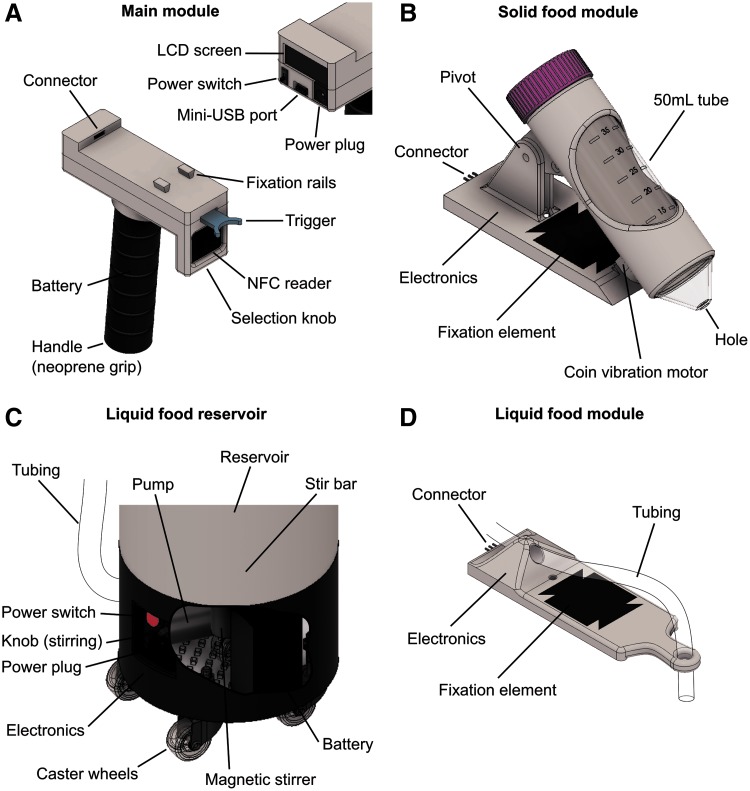
System parts and localization of relevant elements. **(A)** Main module. *Inset*: view of the back of the main module. **(B)** Solid food module. **(C)** Zoom and partial inside view of the skirt of the liquid food reservoir. **(D)** Liquid food module.

The dispenser is composed of three modules (Fig. 1A): a *main module*, a *solid food module* for dry powders, and grains and a *liquid food module* for live microorganisms in water. The main module has an ergonomic handle, a trigger, fixation rails to attach the other modules, and a microcontroller to interface a LCD screen, rotary encoder (to select the mode of operation and navigate in the settings menu; Supplementary Tables S2 and S3), NFC read/write card, and high-power LED (Fig. 2A). The trigger has been designed to fit on Tecniplast tanks, but it can be easily commuted to fit other types of tanks. The main module always acts as the master device while other modules are working devices that respond to the input of the master device.

The solid food module has a removable reservoir (standard 50 mL tube, Falcon ref. 352070) drilled at the tip and mounted on a sheath with a vibration coin motor (Fig. 2B). During normal handling only minute amounts of powder smear from the reservoir, but under vibration a regular flow of grains instantaneously establishes (Supplementary Movie S1). This phenomenon has been previously described^15^ and, in essence, relies on the fluidization of the granular bed by a constant injection of energy to overcome friction among grains. Vibrations are similar to those of video games paddles, and are neither unpleasant nor dangerous for the operator.

The liquid food reservoir (8 L) is attached onto a custom skirt with caster wheels (Fig. 2C). The skirt comprises a battery, pump, and magnetic stirrer. The latter is essential for ensuring homogeneity in the solution and *Artemia* nauplii survival during the whole feeding process. The reservoir can be closed with a lid or left open, and remains at atmospheric pressure at all times. The pump is triggered by a signal coming from the main module and sends the liquid to the module's base for delivery (Fig. 2D).

### Modules construction

Modules have been designed with Fusion 360 (Autodesk). Custom mechanical parts have been 3D-printed, mostly with polylactic acid (PLA) (on an Anet A8 printer) and for some parts in polyethylene terephtalate glycol (PETG) (on a Prusa Mk3 printer). The lid of the liquid reservoir's skirt has been laser-cut (5 mm polymethyl metacrylate (PMMA) on a Full Spectrum Laser Hobby machine). Mechanical assembly has been realized with M3 metallic threaded inserts.

The electronics was custom-made and based on inexpensive, well-documented microcontrollers (Arduino Nano v3.1). For the liquid food module, a dedicated Printed Circuit Board (PCB) has been designed (Eagle, Autodesk) and manufactured (PCBWay) to reduce the footprint, avoid mistakes during soldering and ease mounting. The system is able to detect which module is mounted by means of a resistance specific to each module that creates a voltage divider (Supplementary Data; Supplementary Table S1).

### Calibration

A dedicated setup has been developed for calibrating the solid and liquid food modules, comprising a rigid arm holding the dispenser and a scale (OHAUS PA2102C) as illustrated on Supplementary Figure S1. Both the dispenser and the scale where computer-controlled, and delivered amounts were recorded during series of activation (Supplementary Movies S1 and S2 Supplementary Figures 2 and 3). The same system was used to determine the system's accuracy with runs of 50 trials of random duration, similar to the human accuracy tests.

### Fish room testing

The system has been tested in the aquatic facility of IBPS (*Sorbonne Université*, ∼1.100 tanks, 15.000 fish). The experiments were made in agreement with the European Directive 210/63/EU on the protection of animals used for scientific purposes, and the French application decree *Décret 2013-118*. The aquatic facility has been approved by the French *Service for animal protection and health*, with the approval number A-75-05-25. The cleaning procedure is described in the Supplementary Data.

A simple, preliminary prototype for dispensing solid food with vibration, which was nonmodular, without visual feedback and without NFC reader has been routinely used from September 2017 to September 2018. The final version of the system has been used on a daily basis since September 1, 2018.

**FIG. 3.**
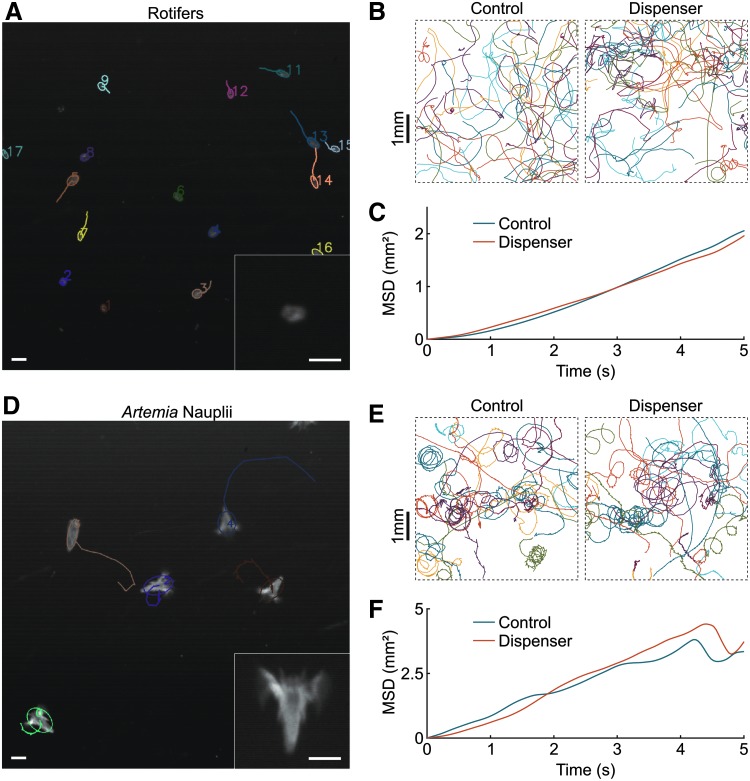
Estimating microorganisms motility after passing through the dispenser. **(A)** Tracking of a movie of rotifers under a binocular. *Inset*: blow-up of a single rotifer. Scale bars: 100 μm. **(B)** Trajectories and **(C)** MSD of moving rotifers in the control condition and just after delivery with the dispenser. **(D)** Tracking of a movie of *Artemia* nauplii under a binocular. *Inset*: blow-up of a single nauplius. Scale bars: 250 μm. **(E)** Trajectories and **(F)** MSD of moving *Artemia* nauplii in the control condition and just after delivery with the dispenser. MSD, mean square displacement.

We observed under a binocular both rotifers (*Rotifera*) and brine shrimps (*Artemia Salina*) before (control) and after passing through the system (reservoir, pump, tubing). Their respective survival rates were estimated with visual inspection and manual counting at different location. We used a camera mounted on the binocular to record movies of the microorganisms dynamics at standard video rate (25 Hz). The movies were processed to extract individual trajectories (Fig. 3, Supplementary Movies S3 and S4) with a novel cross-species tracking software (*FastTrack*) that will be published elsewhere.

### Human accuracy tests

Human subjects were composed of two groups: staff of the fish facility who feed zebrafish more than twice a month (trained group, *n* = 6) and people not working in a fish facility selected at random in the population (random group, *n* = 27). Subject from the random group declared not to suffer from a musculoskeletal disorder. All subjects were aged between 18 and 62 and had no information about the setup or the purpose of the experiment beforehand, except that it would last ∼30 min. Experiments were not remunerated.

The tests were performed in a dedicated room containing only a table with the setup and a chair (Supplementary Fig. S5). The program controlling the screen, scale, and button has been custom made and written in C++ (Qt 5.8). The scale (OHAUS PA2102C) was controlled *via* a serial RS-232 connection and was blinded in a black box such that the subject could not see the LCD screen of the scale. The button was a red pushbutton (normally open switch without latch) mounted on a black plastic box containing a microcontroller (Arduino Nano v3.1) and linked to the computer *via* USB. The powder (Sucrose, 84097-250G; Merck) was disposed in a beaker with a small spoon. The liquid (water with a blue dye, Indigo Carmine, 57000-100G-F; Merck) was disposed in a 500 mL wash bottle. A supplementary 250 mL bottle was also provided in case the subject had to refill the wash bottle during the course of the experiment.

### Analysis

All data from the system calibration setup, the fish room tests and from accuracy tests have been processed with custom scripts in Matlab (R2018a; The MathWorks).

## Results

Operation of the system in a fish room is presented in Supplementary Movies S1 and S2.

### Live food survival

We estimated the survival rates of two standard live feeds, rotifers and *Artemia* nauplii. In control solutions, all animals were moving normally (Fig. 3B, E). After going through the system equipped with the liquid food module, all rotifers were moving in a similar fashion (Fig. 3B) and their observed survival rate was 100%. For *Artemia* it appeared that 5% to 10% of the nauplii died during delivery, probably crushed while passing through the pump. In addition, 20% to 25% of the nauplii were stunned and stopped moving for a few tens (packets of 10 seconds) before resuming normal motion. Trajectories of moving animals were very similar to control (Fig. 3E). We further quantified the motion of rotifers and *Artemia* nauplii before (control) and after delivery by plotting the mean square displacement (MSD) over all trajectories as a function of time (Fig. 3C, F). This is a classical way of characterizing a diffusive process^16^: a straight line indicates diffusive motion, and the slope is equal to four times the diffusion coefficient. The coincidence of the MSD curves in both conditions for rotifers and brine shrimps indicates that the dispenser had no measurable effect on the dynamics of the motile microorganisms, when compared with control.

### Comparison of human and machine accuracy

We developed a psychophysics setup (Supplementary Fig. S5) to measure the average accuracy of humans in tasks mimicking those routinely performed in fish room for manual feeding. Subjects were instructed to enter the test room alone, close the door, sit down, and follow instructions on the screen. All steps of the protocol are detailed in the Supplementary Data. The subjects had to perform successively four parts with 50 trials each, preceded by short training phases of three trials. Parts consisted of (1) pressing a button for a given duration with immediate visual feedback, (2) pressing a button for a given duration without visual feedback, (3) delivering given amounts of powder with a spoon, and (4) delivering given amounts of liquid with a wash bottle. These four tasks encompass all reasonable scenarii for manual or manually assisted delivery. In all tasks the subjects were asked to deliver random integer quantities between 1 and 50, corresponding to a number of animals in arbitrary units (Supplementary Data).

**FIG. 4.**
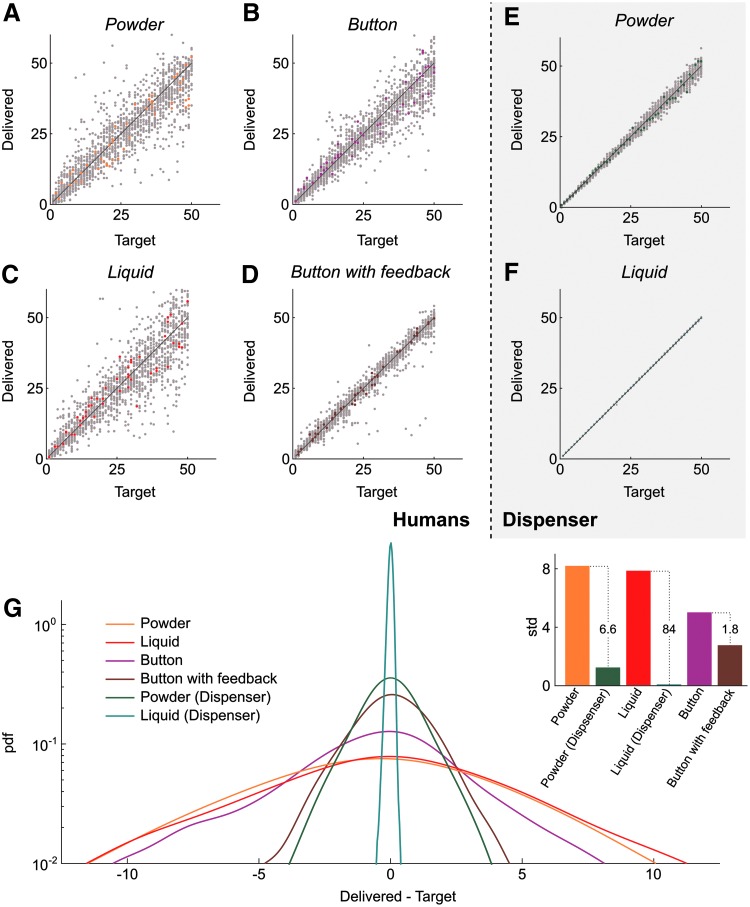
Comparison of human and dispenser accuracy. (**A**–**D**) Quantity delivered by humans as a function of the target quantity in the four tested conditions: powder **(A)**, button **(B)**, liquid **(C)**, and button with visual feedback **(D)**. Data points from all subjects (*n* = 33) are shown in *gray* and data from one individual chosen at random are highlighted in *color*. (**E**, **F**) Quantity delivered by the dispenser as a function of the target quantity for powder **(E)** and liquid **(F)** media. Data points from an equal number of runs (*n* = 33) are show in *gray* and one randomly chosen run is highlighted in *color*. **(G)** pdf of the difference between delivered and target quantities for the different conditions with humans and dispenser. *Inset*: std. Additional numbers on *dotted lines* indicate std ratios. pdf, probability density functions; std, standard deviations.

Experiments on humans revealed a generally poor accuracy (Fig. 4A–D). Surprisingly, we observed no difference between the performance of the trained and random groups, so we pooled all the data for analyses. Errors, quantified as the delivered amount minus the target amount, were symmetrical and Gaussian-distributed (Fig. 4G) with a dispersion that had little dependence on the target amount. The standard deviation of errors with liquid and solid were very high with 7.8 and 8.2 individuals respectively. These values can be considered too large for research-grade rearing; indeed, undernutrition may lead to developmental issues and fertility losses while overfeeding rapidly degrades water quality. Subjects performed slightly better in measuring time with a button, presumably because the mechanical action is reduced to its minimum, with a standard deviation of 5.0 individuals. Having a visual feedback of the elapsed time further improved accuracy, with a standard deviation of 2.8 individuals. Though this is presumably the best accuracy human subjects can achieve, transposed in a fish room that would require that the operator focuses on a screen while feeding.

In our system, NFC detection allows for the dispenser to know the number of animals in the tank and calculate the delivery time accordingly. The operator thus only manages the correct placement of the dispenser over the tank, and the accuracy is solely set by the dispenser's own reproducibility. The latter is excellent (Fig. 4E, F) and the standard deviation of errors goes down to 1.24 individuals for the solid food module and as low as 0.093 individuals for the liquid food module (Fig. 4G), yielding respectively 7-fold and 84-fold decreases in standard deviation when compared to human performance for similar tasks.

### Feeding duration

We measured the average time to feed a complete rack *via* manual feeding (wash bottle for liquid, seed sower for solid food) and semi-automatic feeding in NFC mode (Supplementary Table S4). For inexperienced people, the semi-automatic dispenser slightly increased the feeding time (average +7.5%) with solid food and decreased the feeding time (average −21%) for liquid food. For trained staff, we observed a systematic increase of duration with the semi-automatic dispenser (average +56% for both solid and liquid food).

## Discussion

Our semi-automatic dispenser is a new solution to the numerous issues raised by the pivotal but tedious task of feeding in fish rooms. It is more advanced and convenient than other semi-automatic solutions we are aware of (some being published,^17^ but most are not): it is versatile, reliable, accurate, truly portable, easy to clean, and do not soot over time. It has also several assets when compared to the commercial fully-automatic solution since it is low-cost, low-footprint, and let the operator at the center of the feeding process such that discrepancies are immediately detected and corrected. Without the burden of constantly measuring quantities the operator can use the feeding time to perform routine visual inspection of fish health or check tanks labels. It is also readily accessible to untrained operators, such as students or seasonal staff, who can feed immediately and without any loss of accuracy.

While developing our system it appeared that keeping a high survival rate with a constant flow at high troughtput is a technical challenge. We achieved 100% survival with rotifers but for the bigger *Artemia* only 90% to 95% survival while stunning up to 25% of the nauplii. In practice, Zebrafish ingest inert nauplii as well and no waste is left after a few minutes. Dead and stunned *Artemia* should in principle sink to the bottom of the tank but the tumult created by fish agitation in presence of food scatters the nauplii everywhere in the tank. Careful observation of fish behavior during feeding of *Artemia* with the liquid food module make us suggest that such a 3:1 mixture of mobile and inert nauplii could be *in fine* beneficial to the fish, as they are forced to search for food in various places of the tanks and may adopt richer hunt strategies regarding competition with mates.

We also quantified that our system's accuracy is well suited for animal rearing down to the single-animal scale. Yet some sources of inaccuracy remain: for liquid feed the fluctuations in microorganism concentration in the initial solution are the main source of inaccuracy, while for powders errors come from minute irregularities in the flow rate. We also expect that for powders the reproducibility is highly sensitive to hygrometry, and we checked that the reservoir tube has to be tightly fixed to the sheath, unless large errors can appear (Supplementary Fig. S4).

We finally measured the total feeding time and though there is no noticeable difference for naive individuals when compared to manual feeding, trained staff spent on average +56% more time per rack. This rise can be explained by the fact that trained staff have naturally developed stereotyped gestures over the years for optimizing their manual feeding time. These stereotyped gestures are not desirable in general, since they generate and aggravate musculoskeletal disorders. Also, the longer time spent in front each rack is partly compensated by the consequent reduction of food-filling episodes: the liquid reservoir has a maximal volume equal to 16 wash bottles and the tubes containing solid food can be carried along and loaded very rapidly.

Though our system is now mature enough to be used routinely in a fish room, there is still room for improvements. Modification of the tanks shapes for an easy docking of the semi-automatic feeder could significantly reduce the total feeding time. Another line of research could be to embed a system to automatically count fish in each tank with an embedded camera—for instance, following the work of Silvério *et al.*^18^ That would remove the maintainance of NFC tags on the tanks, but certainly requires an in-depth change of the current design and extensive testing.

In our opinion, one of the most important aspect of this work is the leap on the ground of musculoskeletal disorders. Some stress is still present while handling our dispenser (∼270 and 220 g with and without a module set up) with repeated gesture, especially as it has to be carried at a wide range of heights. Estimating how this can lead to potential injuries is complicated as it depends on the own height and precise movements of each person. However, as soon as the system has been introduced in the fish room, all our staff members observed a relief in forearm pains during and after feeding. A staff member who was previously unable to feed due to repeated wrist tendinitis is now able to feed again without particular pain. These preliminary observations are still to be confirmed over time and with more users, but it is already very promising and we hope that this work will inspire other faculties and companies to invest in research for improving staff health. One possible way for future research is to relieve efforts on shoulder and elbow with an exoskeleton.^19,20^

## Supplementary Material

Supplemental data

Supplemental data

Supplemental data

Supplemental data

Supplemental data
