# Supplementary material for: A Semi-Automatic Dispenser for Solid and Liquid Food in Aquatic Facilities
Source: Zebrafish. 2019 Aug 1;16(4):401–7. doi: 10.1089/zeb.2019.1733 (PMC6685217; doi:10.1089/zeb.2019.1733)
Supplement: Supplemental data [file SupplementaryMaterial.docx]

Supplementary materials

A semi-automatic dispenser for solid and liquid food in aquatic facilities

*Raphaël Candelier, Alex Bois, Stéphane Tronche, Jéremy Mahieu, Abdelkrim Mannioui*

Table of Contents

[1 General information 2](#_Toc536385490)

[1.1 Sources of the prototype 2](#_Toc536385491)

[1.2 Sources of the psychophysics experiment 2](#_Toc536385492)

[1.3 Electronics 2](#_Toc536385493)

[1.4 Operation and settings 3](#_Toc536385494)

[1.4.1 General operation 3](#_Toc536385495)

[1.4.2 Menu tree 4](#_Toc536385496)

[1.4.3 EEPROM management 4](#_Toc536385497)

[1.4.4 Nomenclature of the NFC tags: number of animals and cleaning protocol 5](#_Toc536385498)

[2 Measuring the system’s accuracy 6](#_Toc536385499)

[2.1 Liquid food module 6](#_Toc536385500)

[2.2 Solid food module 7](#_Toc536385501)

[3 Feeding duration 8](#_Toc536385502)

[4 Measuring Human accuracy 9](#_Toc536385503)

[4.1 Rationale and setup 9](#_Toc536385504)

[4.2 Step-by-step description 10](#_Toc536385505)

[5 Supplementary Movies 14](#_Toc536385506)

[5.1 Supplementary Movie 1 14](#_Toc536385507)

[5.2 Supplementary Movie 2 14](#_Toc536385508)

[5.3 Supplementary Movie 3 14](#_Toc536385509)

[5.4 Supplementary Movie 4 14](#_Toc536385510)

# General information

## Sources of the prototype

A patent covering the present semi-automatic feeding system has been filed at the French *Institut National de la Propriété Industrielle* (INPI) and extended for international protection. Source materials including plans, electronic diagrams and Arduino microcode are available upon signature of a material transfer agreement.

## Sources of the psychophysics experiment

The source code of the program used for human accuracy experiments is available under the terms of the CeCILL licence for French regulation and more generally the terms of the GNU General Public License.

All files are available at the following URL:

<http://www.raphael.candelier.fr/Papers/SeAFooD/ReproPsycho.zip>

## Electronics

All parts of the system, including the electronics, have been designed to be accessible and customizable by non-specialists. All components have a through-hole packaging and only basic soldering equipment is required for assembly. For simplicity, we choose the Arduino Nano v.3.1 as microcontrollers for the main and liquid food modules. A PCB (*Printed Circuit Board*) has been designed for housing the electronic components of the liquid food module, in the form of a cape for the Arduino Nano.

Modules are automatically recognized when connected to the main module. A resistance is located in each module between the ID and GND pins, which forms a voltage divider with the internal resistance of the A0 analog input pin of the Arduino. According to Arduino specifications this internal resistance is between 20kΩ and 50kΩ, so the possible resistance ranges are listed below:

| **Resistance value** | **Analog value range** | **Usage** |
| --- | --- | --- |
| 2.2 kΩ | 43 - 102 |  |
| 10 kΩ | 170 – 341 | Solid food module |
| 27 kΩ | 358 – 588 |  |
| 100 kΩ | 682 – 853 | Liquid food module |
| 250 kΩ | 852 - 948 |  |
| ∞ (open circuit) | 1023 | No module detected |

Supplementary Table 1 - Ranges for the recognition resistance and associated analog values and usages. Grayed cells are reserved for future module development.

## Operation and settings

### General operation

Both the liquid and solid food module operate at fixed flow rates. The control over delivered quantities is therefore performed by controlling the delivery times $t_{L}$ and $t_{S}$, which are related to the number of animals $n$ by fixed coefficients $\alpha_{L}$ and $\alpha_{S}$:

- For liquid food: $t_{L}= \alpha_{L}.n$, with by default $\alpha_{L}$ = 80 ms/fish
- For solid food: $t_{S}= \alpha_{S}.n$ with by default $\alpha_{S}$ = 35 ms/fish

Note that both $\alpha_{L}$ and $\alpha_{S}$ have to be adapted to the food type, the feeding protocol and the fish diet: $\alpha_{L}$ depends linearly on the concentration in microorganisms (unless the concentration is very high and change the effective viscosity of the liquid medium, but this is not recommended) while $\alpha_{S}$ depends on the type of solid food and the size of the hole on the delivery tube. The default value of $\alpha_{S}$ has been optimized for grains 300-500µm in diameter (Gemma Wean 0.3) and a hole of 2.8mm. The coefficients can be changed in the settings menu and are kept in memory even when the device is turned off.

When a solid or liquid food module is mounted on the main module, the device automatically detects which module is mounted and chooses which coefficient $\alpha$ to use accordingly.

The system can function in three different modes:

- **Manual mode**. The delivery time is exactly the time during which the trigger is active. The operator thus directly controls the delivered quantity by pulling the trigger for a short or long duration.
- **Fixed mode**. In this mode the amount of delivered quantity is fixed for all tanks. As soon as the trigger is pulled, the device starts to deliver the fixed quantity, regardless of the state of the trigger until delivery is complete. When delivery is over, the system marks a pause of 1 second before being active again. The fixed quantity can be changed in the settings menu. This mode is particularly interesting for tanks with isolated fish ($n$=1) as precise temporal control for one animal is difficult to achieve in manual mode.
- **NFC mode**. In this mode the system has to read first a *Near-Field Communication* (NFC) tag before being active. Once the quantity to deliver is read, it is displayed on the screen and the trigger becomes active. As soon as the trigger is pulled, the device starts to deliver the required quantity, regardless of the state of the trigger until delivery is complete. These steps can be performed in a single movement (see Supplementary Movie 1 and Supplementary Movie 2) since reading the NFC tag is quasi-instantaneous and usually happens before the trigger touches the tank. Once delivery is complete, the system waits to read a different NFC tag before re-activating the trigger.

The operator can easily switch between these three modes with a rotary encoder. The latter is equipped with an RGB LED that shows a different color for each mode (by default: manual=OFF, fixed=cyan, NFC=yellow). The current mode is also displayed on the LCD screen.

Counters of the total number of deliveries (*i.e.* number of tanks) and the total number of fed animals are always displayed on the screen. It is reset to zeros when the device is switched off.

The system is also equipped with a white power LED that illuminates the tanks, allowing the operator *i*) to check that food is actually arriving in the tank and *ii*) to observe the behavior of the animals during feeding. The intensity of this LED can be different at rest and during delivery, and it can be turned off.

Many settings, including the coefficients $\alpha_{L}$ and $\alpha_{S}$, colors and LED intensity can be accessed and modified *via* a menu interface on the main module. When the button on the rotary encoder is pressed for a one second the systems enters in the *Menu mode* (red color of the rotary encoder by default). Navigation in the menu is achieved with the following commands:

- Clockwise / counterclockwise rotation of the rotary encoder: change selection
- Rotary encoder button: Enter, validation
- Trigger: Cancel, go back or exit the menu

### Menu tree

| General | Mode | Manual |  |
| --- | --- | --- | --- |
|  |  | Fixed | *Value* |
|  |  | NFC |  |
|  | Version | *Version* |  |
|  | Reset to default | *Confirmation* |  |
| Liquid | Time per unit | *Value (ms)* |  |
|  | Recognition range | Min | *Value* |
|  |  | Max | *Value* |
| Solid | Time per unit | *Value (ms)* |  |
|  | Recognition range | Min | *Value* |
|  |  | Max | *Value* |
| Lights | Light at rest | *Value (%)* |  |
|  | Light during delivery | *Value (%)* |  |
|  | Colors | Manual | *Color* |
|  |  | Fixed | *Color* |
|  |  | NFC | *Color* |
|  |  | Delivery | *Color* |
|  |  | Menu | *Color* |

Supplementary Table 2: The menu tree, from left to right. Fields in italics are selectable. Color can be either: Red, Green, Blue, Magenta, Yellow, Cyan, White or None.

### EEPROM management

The Arduino Nano has an embed EEPROM memory of 1024 bytes. EEPROM is non-volatile, meaning that it can be used to store settings even when the device is powered off. The following table resumes how the 28 first bytes of EEPROM are managed to store settings:

| **Address bytes** | **Description** | **Possible values** | **Default value** |
| --- | --- | --- | --- |
| 0 | Version first number | 0 – 255 | 1 |
| 1 | Version second number | 0 – 255 | 2 |
| 2 | Version third number | 0 - 255 | 2 |
| 3 | Version day | 0 - 255 | 17 |
| 4 | Version month | 0 - 255 | 01 |
| 5 | Version year, after 2000 | 0 - 255 | 19 |
| 6 | Mode | 0: Manual, 1: Fixed,  2: NFC | 0 |
| 7 | Fixed value (only for the *Fixed* mode) | 0 - 100 | 5 |
| 10 - 11 | Liquid module detection lower value | 0 - 1024 | 680 |
| 12 - 13 | Liquid module detection higher value | 0 - 1024 | 860 |
| 14 – 15 | Solid module detection lower value | 0 - 1024 | 170 |
| 16 - 17 | Solid module detection higher value | 0 - 1024 | 350 |
| 18 - 19 | Coefficient $\alpha_{L}$, milliseconds per animal | 0 – 10,000 | 80 |
| 20 – 21 | Coefficient $\alpha_{S}$, milliseconds per animal | 0 – 10,000 | 35 |
| 22 | LED intensity at rest (%) | 0 – 100 | 1 |
| 23 | LED intensity during delivery (%) | 0 - 100 | 25 |
| 24 | Color at rest, Manual mode | 0: None, 1: White,  2: Red, 3: Green,  4: Blue, 5: Magenta,  6:Cyan, 7: Yellow | 0 |
| 25 | Color at rest, Fixed mode |  | 4 |
| 26 | Color at rest, NFC mode |  | 7 |
| 27 | Color during delivery |  | 1 |
| 28 | Color in the menu |  | 5 |

Supplementary Table 3: Management of EEPROM memory in the main module.

### Nomenclature of the NFC tags: number of animals

Data has been written on the NFC tags with the free version of [NFCTools](https://play.google.com/store/apps/details?id=com.wakdev.wdnfc&hl=fr) and a compatible smartphone (Samsung Galaxy Young 2 SM-G130). The written data is always plain text.

For NFC tags used for feeding, only the number of animals in the tank is written on the tag. So when the main module reads a single number, it is interpreted as a number of animals and the delivery signal is set for the corresponding duration. For instance:

| **18** | Sets delivery for 18 animals |
| --- | --- |

Special diets can be programmed for some tanks only (by punctually changing the value on the NFC tag) or globally (by changing the coefficients $\alpha_{L}$ and $\alpha_{S}$).

The cleaning procedure of the liquid food module is also defined on a separate NFC tag. When the module reads such a tag, it automatically triggers the cleaning. The protocols are composed of sequences of delivery with 10 second pauses in between. The syntax is as follows:

| **1x60** | Triggers a continuous delivery phase lasting 60 seconds |
| --- | --- |
| **3x60** | Triggers a cleaning protocol composed of three 60 seconds-long delivery phases separated by 10 second pauses. |

NFC tags can have many shape and materials, and we chose round 25mm-diameter stickers since they are cheaper (small price differences become significant with thousands of tanks). They do not go well in the cleaning machine, so in order to remove and replace them at will we glued it on transparent post-its (see Figure 1.B-C, Supplementary Movie 1 and Supplementary Movie 2) or on thin plastic film usually used for electrostatic stickers. As the information stored on the NFC tags can be re-written endlessly, it is thus easy to reuse it on clean tanks.

### cleaning protocol

The system has a cleaning procedure to avoid contamination between two successive use. It relies on the joint action of three procedures:

- Long cycles of clean water (3 times 8L during 60 seconds) going through the system remove all microorganisms and debris. We did not quantify the remaining amounts, but no artemia/rotifer could be observed by visual inspection of the rejected water after the first cycle.

- An Additional cycle with Virkon (1%) followed by washing with water cycles can be performed, to sanitize the pump and tubing.

- All tubes can be changed on a regular basis (*e.g.* once a month).

# Measuring the system’s accuracy

We used the setup show in Supplementary Figure 1 to measure the system’s accuracy for both the solid and liquid food modules. The scale (OHAUS PA2102C) was connected to the computer *via* an USB to RS232 serial converter. For the solid food tests we used Gemma Wean 0.3 (Planktovie) in a 50mL tube with a 2.8mm drilled hole, and for the liquid food module we used tap water.


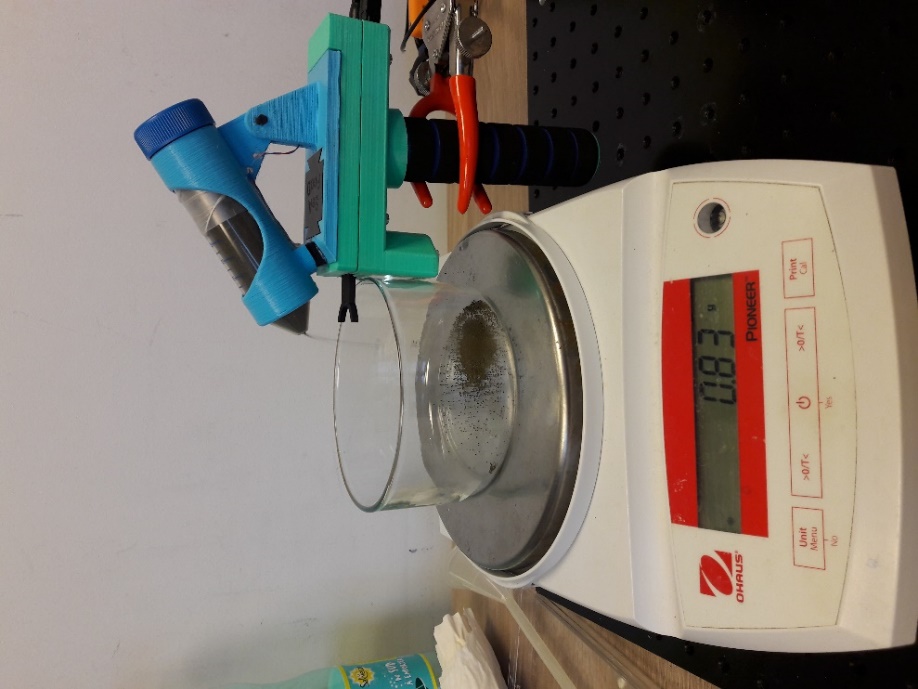

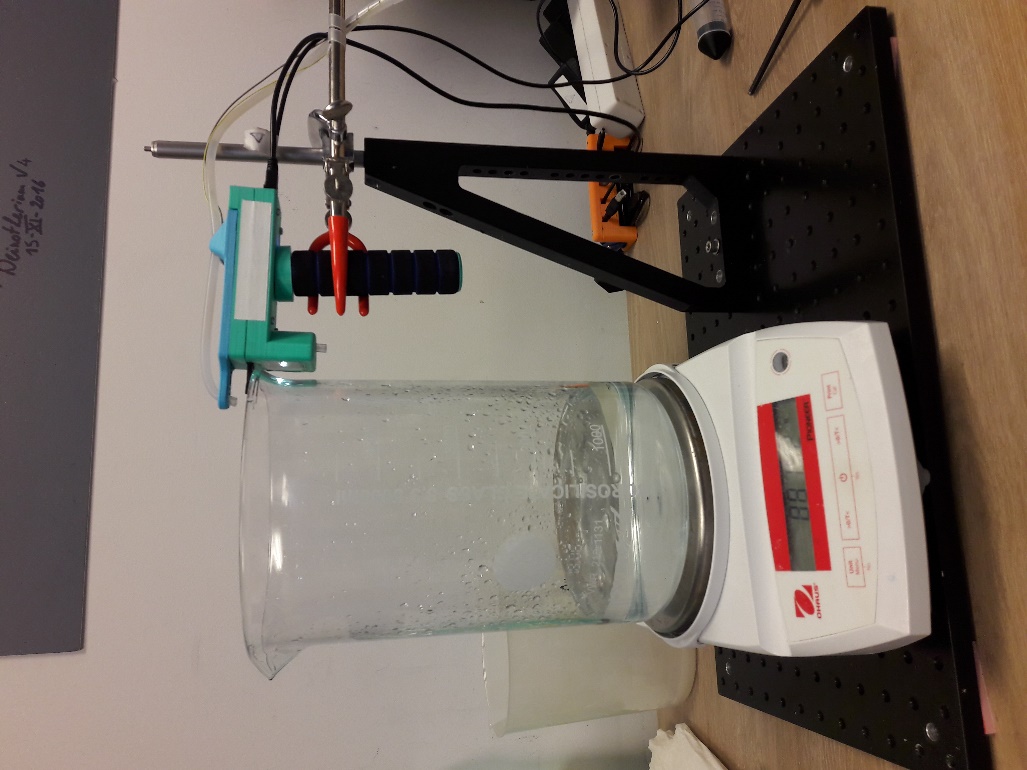


Supplementary Figure 1 – Left: Picture of the setup used to measure quantities delivered by the solid food module. Right: Picture of the setup used to measure quantities delivered by the liquid food module.

## Liquid food module


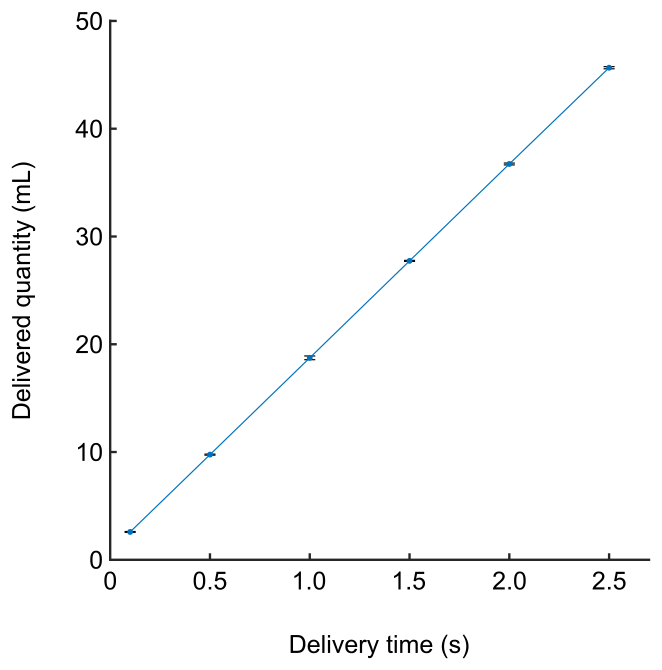


Supplementary Figure 2 – Delivered quantity as a function of the delivering time for the liquid food module. Error bars: standard deviation.

## Solid food module


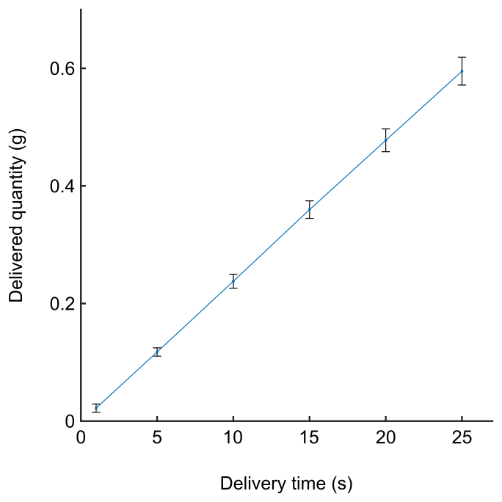


Supplementary Figure 3 - Delivered quantity as a function of the delivering time for the solid food module. Error bars: standard deviation.

In contrast with the liquid food module, several factors can influence the accuracy of the solid food module. For instance, if the containing tube is not tightly attached to the sheath and is able to rotate during vibration the reproducibility is greatly hampered:


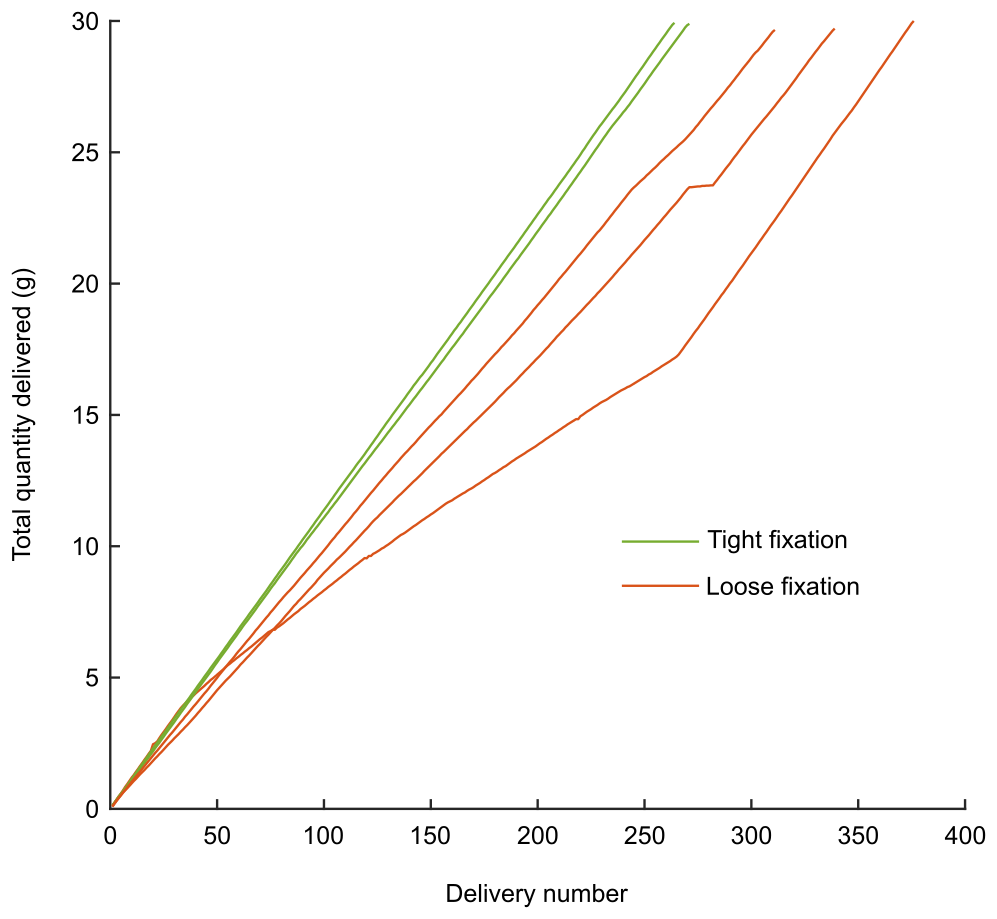


Supplementary Figure 4 – Cumulative quantity of food delivered during runs of 400 trials of 2.0 second of vibration. The containing tube is full (30g) at the beginning of each run and empty at the end. Tight (green) and loose (red) fixation of the containing tube inside the sheath are compared.

Also, controlling the degree of moisture in the powder is essential for accuracy. Water creates bonds between grains that may lead to large aggregates. The latter need a lot of energy to break, so the granular bed is vibration-fluidized with reduced efficiency and severe losses of efficiency can appear. So the food should be kept as dry as possible, which may be difficult in a fish room. We recommend to store the food in a dry place (room with controlled hygrometry or drying box with desiccant).

# Feeding duration

|  |  | Fish room staff | Unexperienced people |
| --- | --- | --- | --- |
| Solid food | Manual (seed sower) | 45.6 | 82.7 |
|  | Dispenser (NFC mode) | 71.6 | 89.0 |
| Liquid food | Manual (wash bottle) | 41.2 | 112.5 |
|  | Dispenser (NFC mode) | 64.2 | 88.9 |

Supplementary table 4 – Average feeding time (seconds) for rack of 600 fish with an average of 15.4 fish/tank. Fish room staff have fed manually for many years, and used the semi-automatic dispenser for 4 months before the test. Unexperienced operators were volunteer students who had never fed before. Students were shown typical quantities to deliver by trained staff, and they could practice on a few tanks to adjust delivered quantities just before the test. For each condition, 3 subjects were tested on two racks.

# Measuring Human accuracy

## Rationale and setup

Our system has a good accuracy over delivered quantities, which we could measure with the automated setup shown in Supplementary Figure 1. But to determine if there is a gain as compared to manual feeding, we had to measure the accuracy of humans in standard feeding tasks. We thus developed a psychophysics setup (Supplementary Figure 5) to measure the average accuracy and reproducibility of human subjects in delivering some liquid with a washer bottle and some powder with a spoon. We also tested human reproducibility on a time-measuring task during which the operator had to press a button for a given amount of time, with or without visual feedback on the elapsed time.

The button was fixed on a small black enclosure containing an Arduino Nano, which was connected to the computer *via* USB. The scale (OHAUS PA2102C) was connected to the computer *via* a USB to RS232 serial converter. The powder was sucrose (Merck 84097-250G) and the liquid was distilled water with a blue dye (Indigo carmine, Merck 57000-100G-F).

Subjects were always asked to deliver an integer quantity between 1 and 50. We used arbitrary units in all parts of the experiment, and the following coefficients (unknown to the subject) were used:

- One unit of time was 100ms for tasks with the button
- One unit of mass was 60mg for the solid delivery task
- One unit of mass was 400mg (400µL) for the liquid delivery task


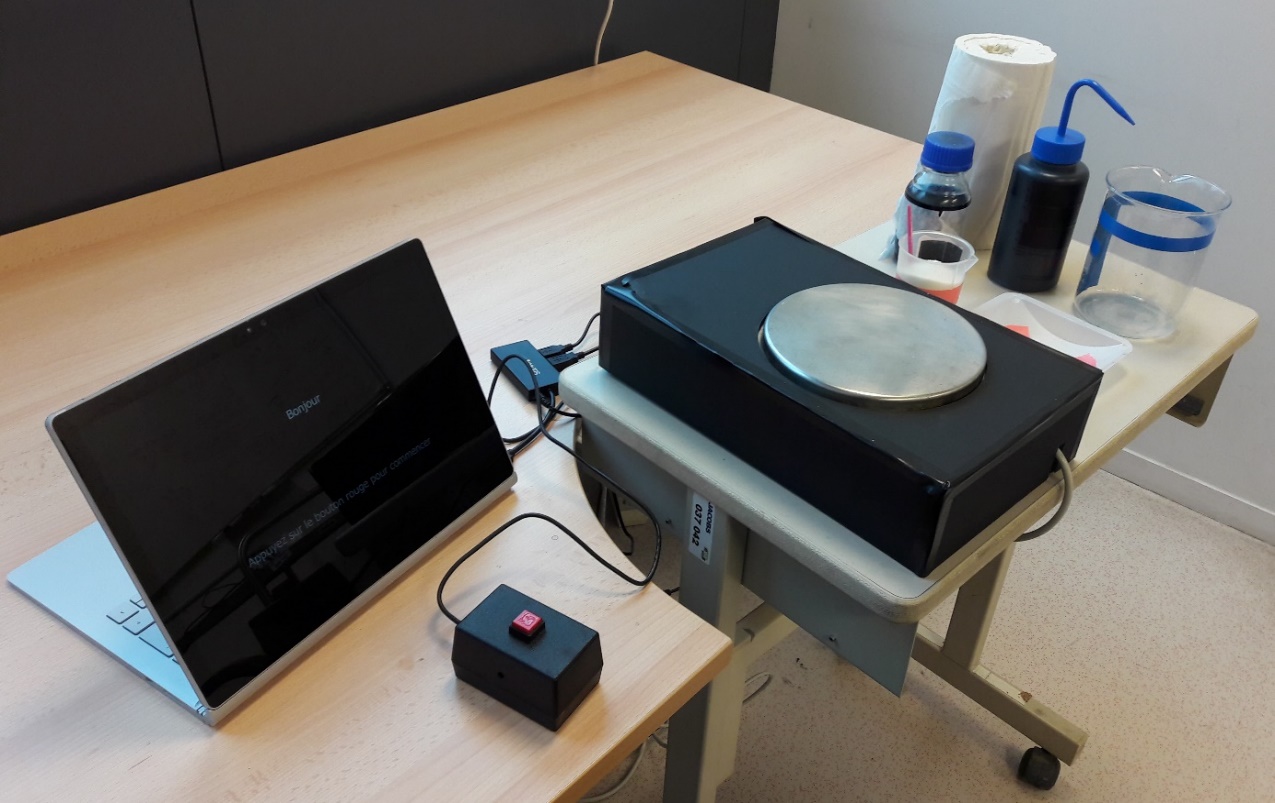


Supplementary Figure 5: Setup for testing human accuracy in measuring amounts of powder, liquid and time. From left to right: screen, button, scale, powder recipient with spoon (pink), liquid wash bottle.

## Step-by-step description

|  | **Step-by-step display (French)** | **English translation** |
| --- | --- | --- |
|  | Bonjour  Appuyez sur le bouton rouge pour commencer | Hello  Press the red button to start |
|  | *Wait for button press* | |
|  | Cette expérience va se dérouler en trois parties de durée égale.  Dans chaque partie vous pouvez utiliser  n'importe quelle main et en changer à tout moment.  Appuyez sur le bouton pour continuer | This experiment is composed of three parts of equal duration  In each part you can use either your left or right hand and change anytime.  Press the button to continue |
|  | *Wait for button press* | |
|  | Dans la première partie vous allez utiliser  uniquement ce bouton rouge.  Vous devrez appuyer sur le bouton pendant une durée donnée en essayant d'être le plus précis possible: pas trop court, pas trop long.  Le temps se déroule dans une unité arbitraire,  et nous allons tout d'abord nous familiariser avec.  Appuyez sur le bouton pour continuer | In the first part you will only use  this red button.  You will have to press the button for a given duration and try to be as accurate as possible:  not too short, not too long.  Time is counted in an arbitrary unit, and we will first familiarize with it.  Press the button to continue |
|  | *Wait for button press* | |
|  | Appuyez sur le bouton et restez appuyé  Observez le compteur défiler  jusqu'à ce que vous relachiez  - | Press and hold the button  Observe the counter  until you release the button  - |
|  | *When the button is pressed the – is replaced by numbers linearly increasing with time. When the button is released, the program holds the display for one second.* | |
|  | Maintenant essayez de faire exactement 18  - | Now try to make exactly 18  - |
|  | *When the button is pressed the – is replaced by numbers linearly increasing with time. When the button is released, the program holds the display for one second.* | |
|  | Pas facile, mais vous vous en sortez bien.  Essayez de faire exactement 23  - | Not easy, but you are doing well.  Try to make exactly 23  - |
|  | *When the button is pressed the – is replaced by numbers linearly increasing with time. When the button is released, the program holds the display for one second.* | |
|  | Maintenant essayez de faire exactement 7  - | Now try to make exactly 7  - |
|  | *When the button is pressed the – is replaced by numbers linearly increasing with time. When the button is released, the program holds the display for one second.* | |
|  | Cet entrainement est terminé,  nous allons maintenant passer à la phase de test.  La tâche reste la même, vous devez appuyer  pendant le temps indiqué, exactement.  Appuyez sur le bouton pour commencer | This training is over,  We shall now pass onto the test phase.  The task remains the same, you have to press  the button for the indicated time, exactly.  Press the button to start |
|  | *Wait for button press* | |
| Repeat 50 times | Vous devez atteindre exactement  *N*  - | You have to reach exactly  *N*  *-* |
|  | N *is a random integer between 1 and 50, different from the previous one.*  *When the button is pressed the – is replaced by numbers linearly increasing with time. When the button is released, the program holds the display for one second.* | |
|  | Maintenant nous allons augmenter un peu la difficulté  et vous ne verrez plus le compteur défiler.  Le résultat s'affichera  une fois que vous relacherez le bouton.  Essayez de faire exactement 25.  - | Now we will increase a little bit the difficulty  and you won’t see the counter anymore.  The result will appear only  once you release the button.  Try to make exactly 25.  - |
|  | *When the button is pressed time is measured but not displayed. When the button is released, the – is replaced by the elapsed time and display is held for 1.5 second.* | |
| Repeat 50 times | Vous devez atteindre exactement  *N*  - | You have to reach exactly  *N*  *-* |
|  | N *is a random integer between 1 and 50, different from the previous one.*  *When the button is pressed time is measured but not displayed. When the button is released, the – is replaced by the elapsed time and display is held for 1.5 second.* | |
|  | La première partie est maintenant terminée.  A partir de maintenant le bouton ne sert plus  qu'a avancer dans les instructions.  Appuyez sur le bouton pour continuer | The first part is now over.  From now on the button will only be used  to go forward in the instructions.  Press the button to continue |
|  | *Wait for button press* | |
|  | Prenez la coupelle de pesée avec une croix rose  et déposez-la sur la balance.  Appuyez sur le bouton quand la coupelle est en place | Take the weighing dish with a pink cross  and place it on the scale.  Press the button when the dish is placed |
|  | *Wait for button press* | |
|  | Dans la deuxième partie vous devez verser  une quantité donnée de poudre dans la coupelle:  pas moins, pas trop.  La poudre se trouve dans le petit becher rose.  Vous devez utiliser la cuillère rose fournie,  et ne faire qu'un seul versement par consigne.  Vous ne devez pas toucher la table pendant l'expérience.  Appuyez sur le bouton pour continuer | In the second part you will have to verse  a given quantity of powder in the dish:  not less, not more.  The powder is in the small pink beaker.  You shall use the pink spoon,  and verse powder only once per order.  You shall not touch the table during the experiment.  Press the button to continue |
|  | *Wait for button press* | |
|  | Ici encore, la quantité de poudre est comptée  en unité arbitraire  Déposez une demi-cuillère de poudre.  Il faut attendre que la balance se stabilise  après chaque versement.  - | Here again, the amount of powder is counted  in arbitrary units  Sprinkle half a spoon of powder.  You have to wait for the scale to stabilize  after each trial.  - |
|  | *When the scale stabilizes with a positive weight difference the – is replaced by the measured quantity and display is held for 1.5 second.* | |
|  | Maintenant essayez de faire exactement 18  - | Now try to make exactly 18  - |
|  | *When the scale stabilizes with a positive weight difference the – is replaced by the measured quantity and display is held for 1.5 second.* | |
|  | Pas facile, mais vous vous en sortez bien.  Essayez de faire exactement 23  - | Not easy, but you are doing well.  Try to make exactly 23  - |
|  | *When the scale stabilizes with a positive weight difference the – is replaced by the measured quantity and display is held for 1.5 second.* | |
| Repeat 50 times | Vous devez atteindre exactement  *N*  - | You have to reach exactly  *N*  *-* |
|  | N *is a random integer between 1 and 50, different from the previous one.*  *When the scale stabilizes with a positive weight difference the – is replaced by the measured quantity and display is held for 1.5 second.* | |
|  | La deuxième partie est maintenant terminée.  Veuillez enlever la coupelle de pesée  et la mettre de côté, avec le becher rose.  Appuyez sur le bouton pour continuer | The second part is now over.  Please take off the weighing dish  and place it aside, with the pink beaker.  Press the button to continue |
|  | *Wait for button press* | |
|  | Prenez le grand becher bleu en verre  et déposez-le sur la balance.  Appuyez sur le bouton quand le becher bleu  est en place | Take the large glass blue beaker  and place it on the scale.  Press the button when the blue beaker  is placed |
|  | *Wait for button press* | |
|  | Dans la troisième partie vous devez verser  une quantité donnée de liquide dans le becher:  pas moins, pas trop.  Comme précédemment, vous n'avez droit qu'à  un seul versement par consigne.  Appuyez sur le bouton pour continuer | In the third part you will have to verse  a given quantity of liquid in the beaker:  not less, not more.  As previously, you are only allowed to  deliver once per order.  Press the button to continue |
|  | *Wait for button press* | |
|  | La quantité de liquide est mesurée en unité arbitraire.  Effectuez une pression sur la pissette bleue.  Il faut attendre que la balance se stabilize  après chaque versement.  - | The amount of liquid is measured in arbitrary units.  Press once on the blue washer bottle.  You have to wait for the scale to stabilize  after each dump.  - |
|  | *When the scale stabilizes with a positive weight difference the – is replaced by the measured quantity and display is held for 1.5 second.* | |
|  | Maintenant essayez de faire exactement 18  - | Now try to make exactly 18  - |
|  | *When the scale stabilizes with a positive weight difference the – is replaced by the measured quantity and display is held for 1.5 second.* | |
|  | Pas facile, mais vous vous en sortez bien.  Essayez de faire exactement 23  - | Not easy, but you are doing well.  Try to make exactly 23  - |
|  | *When the scale stabilizes with a positive weight difference the – is replaced by the measured quantity and display is held for 1.5 second.* | |
| Repeat 50 times | Vous devez atteindre exactement  *N*  - | You have to reach exactly  *N*  *-* |
|  | N *is a random integer between 1 and 50, different from the previous one.*  *When the scale stabilizes with a positive weight difference the – is replaced by the measured quantity and display is held for 1.5 second.* | |
|  | Cette expérience est maintenant terminée.  Merci d'y avoir participé.  Vous pouvez quitter la pièce. | This experiment is now over.  Thanks for participating.  You can leave the room. |

# Supplementary Movies

## Supplementary Movie 1

**Solid food module**. 1) Mounting of the solid food module (blue and black) on the main module (cyan). 2) Calibration setup, during a calibration experiment. The system is computer-controlled and delivers for random, uniformly distributed durations while recordings are performed with a scale. 3) Close-up view of the hole upon delivery. 4) Delivery in a tank of a fish room, in NFC mode. 5) Workflow on successive tanks.

## Supplementary Movie 2

**Liquid food module**. 1) Mounting of the liquid food module (blue and black) on the main module (cyan). 2) Calibration setup, during a calibration experiment. The system is computer-controlled and delivers for random, uniformly distributed durations while recordings are performed with a scale. 3) Close-up view of the tube end upon delivery. 4) Delivery in tanks of a fish room, in NFC mode. 5) Workflow on successive tanks.

## Supplementary Movie 3

**Tracking rotifers.** 1) Motion of rotifers in the control condition. 2) Same movie, with tracking overlaid.

## Supplementary Movie 4

**Tracking *Artemia* nauplii.** 1) Motion of brine shrimps in the control condition. 2) Same movie, with tracking overlaid.
